# Marinopyrrole Derivatives with Sulfide Spacers as Selective Disruptors of Mcl-1 Binding to Pro-Apoptotic Protein Bim

**DOI:** 10.3390/md12084311

**Published:** 2014-07-29

**Authors:** Chunwei Cheng, Yan Liu, Maria E. Balasis, Thomas P. Garner, Jerry Li, Nicholas L. Simmons, Norbert Berndt, Hao Song, Lili Pan, Yong Qin, K. C. Nicolaou, Evripidis Gavathiotis, Said M. Sebti, Rongshi Li

**Affiliations:** 1 Key Laboratory of Drug Targeting and Drug Delivery Systems of the Ministry of Education and State Key Laboratory of Biotherapy, Department of Medicinal Natural Products, West China School of Pharmacy, Sichuan University, Chengdu 610041, China; E-Mails: chengchunwei666@163.com (C.C.); haoright@163.com (H.S.); pande179@163.com (L.P.); 2 Department of Drug Discovery and Chemical Biology and Molecular Medicine Program, H. Lee Moffitt Cancer Center and Research Institute, 12902 Magnolia Drive, Tampa, FL 33612, USA; E-Mails: yan.liu@unmc.edu (Y.L.); maria.balasis@moffitt.org (M.E.B.); jerry.li@ucsf.edu (J.L.); Norbert.berndt@moffitt.org (N.B.); said.sebti@moffitt.org (S.M.S.); 3 Department of Pharmaceutical Sciences, Center for Drug Discovery, College of Pharmacy, and Cancer Genes and Molecular Regulation Program, Buffett Cancer Center, University of Nebraska Medical Center, 986805 Nebraska Medical Center, Omaha, NE 68198, USA; 4 Departments of Biochemistry and Medicine, Albert Einstein College of Medicine, Jack and Pearl Resnick Campus, 1300 Morris Park Avenue, Forchheimer G46, Bronx, NY 10461, USA; E-Mails: thomas.garner@einstein.yu.edu (T.P.G.); evripidis.gavathiotis@einstein.yu.edu (E.G.); 5 Department of Chemistry and The Skaggs Institute for Chemical Biology, The Scripps Research Institute, 10550 North Torrey Pines Road, La Jolla, CA 92037, USA; E-Mails: nsimmons@scripps.edu (N.L.S.); kcn@rice.edu (K.C.N.); 6 The Innovative Drug Research Center, Chongqing University, Chongqing 400000, China; 7 Department of Chemistry, BioScience Research Collaborative, Rice University, 6500 Main Street, Houston, TX 77030, USA; 8 Department of Oncologic Sciences, Morsani College of Medicine, University of South Florida, 12901 Bruce B. Downs, Tampa, FL 33612, USA

**Keywords:** marinopyrroles, protein-protein interaction disruptors, apoptosis, SAR

## Abstract

A series of novel marinopyrroles with sulfide and sulphone spacers were designed and synthesized. Their activity to disrupt the binding of the pro-apoptotic protein, Bim, to the pro-survival proteins, Mcl-1 and Bcl-xL, was evaluated using ELISA assays. Fluorescence-quenching (FQ) assays confirmed the direct binding of marinopyrroles to Mcl-1. Benzyl- and benzyl methoxy-containing sulfide derivatives **4** and **5** were highly potent dual Mcl-1/Bim and Bcl-xL/Bim disruptors (IC_50_ values of 600 and 700 nM), whereas carboxylate-containing sulfide derivative **9** exhibited 16.4-fold more selectivity for disrupting Mcl-1/Bim over Bcl-xL/Bim binding. In addition, a nonsymmetrical marinopyrrole **12** is as equally potent as the parent marinopyrrole A (**1**) for disrupting both Mcl-1/Bim and Bcl-xL/Bim binding. Some of the derivatives were also active in intact human breast cancer cells where they reduced the levels of Mcl-1, induced programd cell death (apoptosis) and inhibited cell proliferation.

## 1. Introduction

Marinopyrroles were first reported to show antibiotic activity against methicillin-resistant *Staphylococcus aureus* (MRSA) in 2008 by the Fenical group [1]. Due to their novel molecular structures and promising biological properties, marinopyrroles have attracted considerable attention [2,3,4,5,6,7,8,9,10,11,12,13,14,15,16]. Following our finding that (±)-marinopyrrole A (**1**) antagonizes Mcl-1 and overcomes resistance of human cancer cells to the Bcl-xL antagonist ABT-737 [10], we recently reported a series of novel cyclic marinopyrroles as disruptors of protein-protein interactions between the pro-apoptotic protein, Bim, and the pro-survival proteins, Bcl-xL and Mcl-1 [16].

Apoptosis evasion is one of the most important hallmarks that cells must acquire to become cancerous [17,18]. One of the major mechanisms by which cancer cells evade apoptosis is by over expressing Bcl-xL, Bcl-2 and/or Mcl-1 contributing not only to tumorigenesis but also to tumor resistance to chemotherapy [18]. Several small molecule inhibitors of the pro-survival Bcl-2 family of proteins have been identified [19,20,21]. To date, the most extensively studied and promising small molecule BH3 mimetic is ABT-737 or its orally-available ABT-263. However, human tumors that overexpress Mcl-1 are resistant to Bcl-xL/Bcl-2-selective agents such as ABT-737 and ABT-263 [22,23,24]. Fewer Mcl-1 antagonists have been reported, most are not highly selective for Mcl-1 and none have been developed enough to reach clinical trials [25,26,27,28,29,30,31]. Here, we report on the design of a series of marinopyrroles with sulfide and sulphone spacers, some as dual Mcl-1 and Bcl-xL antagonists and others as selective disruptors of Mcl-1 binding to Bim.

## 2. Results and Discussion

### 2.1. Design of Marinopyrrole Derivatives

**Figure 1:**
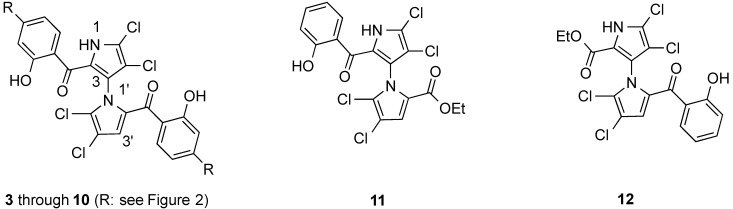
Marinopyrroles with sulfide and sulphone spacers and nonsymmetrical marinopyrroles.

With the success of our synthetic and SAR studies on symmetrical, nonsymmetrical and cyclic marinopyrrole derivatives [3,6,7,14,15,16] and based on our results that marinopyrrole A (**1**) binds to Mcl-1 in two regions according to chemical shift perturbations and docking studies [10], we focused our attention on a series ofsymmetrical derivatives with sulfide and sulphone spacers substituted in the *para-*position relative to the carbonyl group on both aromatic rings (**3** to **10**). We were particularly interested in exploring the structure activity relationships (SARs) of these series of marinopyrroles to probe: (i) whether the molecular geometries play a role; (ii) how large a functional group is tolerated; (iii) whether hydrophobic or hydrophilic groups are preferred; (iv) if hydrogen bond donors or hydrogen bond acceptors are desirable; (v) whether functional groups with ionic interactions are allowed. To evaluate the potential differences in activity between the nonsymmetrical marinopyrroles **11** and **12**, both compounds [5] were included in this study (Figure 1). Some of the marinopyrroles designed for this study are potential candidates for improving aqueous solubility as the parent marinopyrrole A (**1**) exhibited poor solubility which hinders its further development.

### 2.2. Synthesis of Marinopyrrole Derivatives

**Scheme 1:**
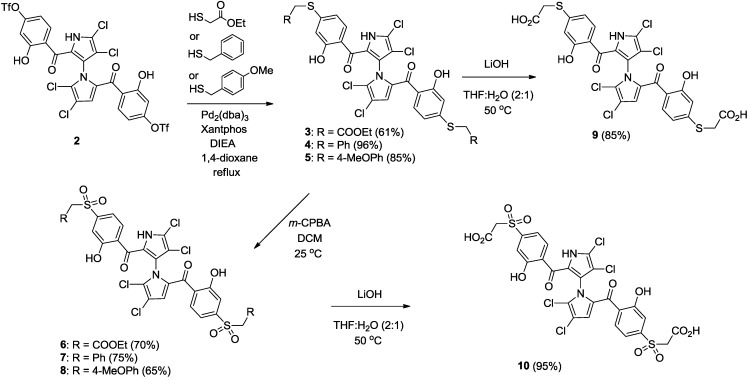
Synthesis of marinopyrroles **3** to **10**.

Starting from our previously reported compound **2** [14] as shown in Scheme 1, palladium-mediated nucleophilic substitution of the triflate **2** with ethyl 2-mercaptoacetate, phenylmethanethiol and (4-methoxyphenyl) methanethiol gave **3** (61%), **4** (96%) and **5** (85%), respectively. Sulfides **3**, **4** and **5** were oxidized to the corresponding sulphones **6** (70%), **7** (75%) and **8** (65%) with *m*-chloroperbenzoic acid (*m*-CPBA). State whether Baeyer-Villiger oxidation by-products are detected. The carboxylic acids **9** and **10** were obtained by saponification of the corresponding esters **3** and **6** using LiOH in 85% and 95% yields.

### 2.3. Physicochemical Properties and SAR of the Marinopyrroles

**Figure 2:**
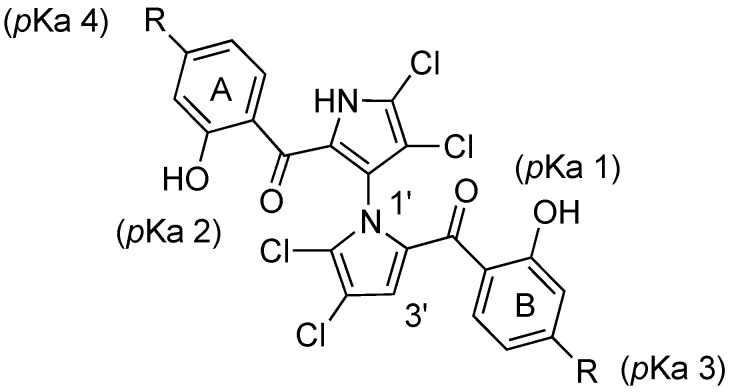
Structure of marinopyrroles.

Both p*K*_a_ and log *p* values were calculated using ChemAxon Software Version 5.12.3 [32,33]. The p*K*_a_ values of marinopyrrole A (**1**) are predicted to be 7.8 (p*K*_a_ 1) and 8.4 (p*K*_a_ 2), respectively (Figure 2 and Table 1). As reported previously [14,15,16], the difference in p*K*_a_ values for the hydroxyl group in ring A and ring B is presumably due to the axially chiral environment. The p*K*_a_ values of **1** are 1.6–2.2 log units lower than that of phenol (p*K*_a_ = 9.98) [34]. An equilibrium may exist between open and closed conformations in **1**, similar to those observed in a recent report of intramolecular hydrogen bonding [35]. The Fenical group reported the X-ray structure of marinopyrrole B (3′-Br analogue of **1**) that indicated the preference for the formation of intramolecular hydrogen bonds between the *peri*-hydroxyl and the carbonyl group [1]. These intramolecular hydrogen bond interactions contribute to increasing not only compound acidity, but also its lipophilicity [35]. The calculated log *p* value of **1** is 5.6, which marginally violates the Rule of Five (RO5), drug-like properties formulated by Lipinski [36]. The calculated p*K*_a_ 1 and p*K*_a_ 2 values of marinopyrroles in Figure 2 range from 6.7 to 8.4. Compound **9** has p*K*_a_ 3 (2.9) and p*K*_a_ 4 (3.5) values, and **10** has a p*K*_a_ 3 (2.2) and p*K*_a_ 4 (2.9) due to the carboxylic acid functional group. Clog *p* values of compounds **9** and **10** are 5.3 and 2.9, respectively. While the former marginally violates the RO5, the latter resides within the suggested range for drug-like compounds. Compound **6** also has a Clog *p* value of 3.7 whereas the remaining compounds **3**, **4**, **5**, **7** and **8** violate RO5 with compounds **4** and **5** being five log unit higher than the desired limit of lipophilicity. Both nonsymmetrical marinopyrroles **11** and **12** have Clog *p* values of 4.5.

**Table 1:** ELISA and physicochemical properties of marinopyrroles.

Compound	Substituent (R)	Mcl-1/Bim ^a^	Bcl-xL/Bim ^a^	p*K*_a_ 1 ^b^	p*K*_a_ 2 ^b^	p*K*_a_ 3 ^b,c^	p*K*_a_ 4 ^b,c^	Clog *p* ^b^
**1**	H	8.9 ± 1.0 ^d^	16.4 ± 3.3 ^d^	7.8	8.4	-	-	5.6
**3**	SCH_2_CO_2_Et	1.8 ± 0.3	1.2 ± 0.2	7.8	8.4	-	-	6.1
**4**	SCH_2_Ph	0.7 ± 0.2	0.6 ± 0.2	7.8	8.4	-	-	10.2
**5**	SCH_2_ (*p-*MeOPh)	0.7 ± 0.1	0.6 ± 0.1	7.8	8.4	-	-	9.7
**6**	SO_2_CH_2_CO_2_Et	37.3 ± 3.1	>100	6.7	7.3	-	-	3.7
**7**	SO_2_CH_2_Ph	7.3 ± 1.4	69.3 ± 15.8	6.7	7.3	-	-	6.9
**8**	SO_2_CH_2_ (*p-*MeOPh)	17.4 ± 3.1	>100	6.7	7.3	-	-	6.4
**9**	SCH_2_CO_2_H	6.1 ± 1.3	>100	7.8	8.4	2.9	3.5	5.3
**10**	SO_2_CH_2_CO_2_H	63.0 ± 5.4	>100	6.7	7.3	2.2	2.9	2.9
**1**	^e^ See Figure 1	25.1 ± 4.7	96.6 (*n* = 2)	-	8.1	-	-	4.5
**12**	^e^ See Figure 1	11.5 ± 1.9	17.6 ± 4.5	8.1	-	-	-	4.5

^a^ IC_50_ in micromolar (average ± SEM, *n* ≥ 3); ^b^ calculated using ChemAxon Software Version 5.12.3; ^c^ p*K*_a_ values from carboxylic acid group; ^d^ ELISA data previously reported by us [16]; ^e^ Compounds were reported previously by us [5].

As reported previously [10,16], the IC_50_ value of racemic marinopyrrole A (**1**) to disrupt the binding of Mcl-1 to Bim was 8.9 μM. The IC_50_ value of **1** to disrupt the binding of Bcl-xL to Bim (16.4 μM) was consistent with our recent report [16] but lower than we originally reported [10], due to the 2.5 times lower Bcl-xL (not Mcl-1) concentration utilized in our present assay (10 nM)* vs.* that originally used (25 nM). Symmetrical marinopyrroles with sulfide spacers (**3**–**5**) are five- to 13-fold and 20- to 27-fold more potent than **1** against Mcl-1/Bim and Bcl-xL/Bim, respectively (Figure 2). The sulfide substitutions greatly increased potency but did not alter selectivity as **3**, **4** and **5** are also dual Mcl-1 and Bcl-xL antagonists (Figure 2). Compounds **4** and **5** are the most potent in the series with IC_50_ values of 0.7 and 0.6 μM against Mcl-1/Bim and Bcl-xL/Bim, respectively. Marinopyrroles with a sulphone spacer (**6**–**8**) are at least 16-fold less active than their sulfide counterparts. This difference is presumably due to different molecular geometries of the –S– and –SO_2_– bonds which might result in desired and undesired orientation of the substituents in the binding pockets. Interestingly, compound **9** demonstrated 16.4-fold selectivity for Mcl-1/Bim over Bcl-xL/Bim with an IC_50_ value of 6.1 μM and >100 μM, respectively. Nonsymmetrical marinopyrrole **12** exhibited similar potencies to **1** against both Mcl-1/Bim and Bcl-xL/Bim although another nonsymmetrical marinopyrrole **11** is much less active than the parent marinopyrrole **1** against Mcl-1/Bim and Bcl-xL/Bim.

### 2.4. Direct Binding Measurement by Fluorescence Quenching

**Figure 3:**
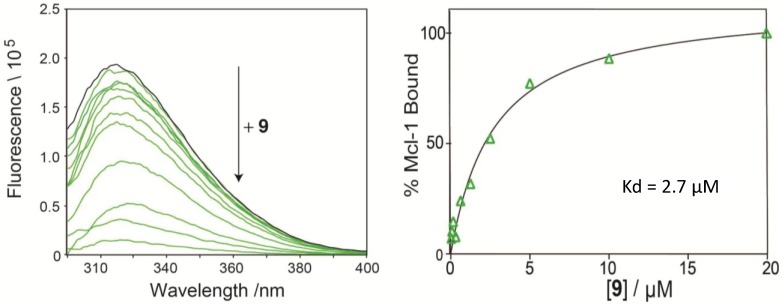
Direct binding of **9** to Mcl-1 measured by fluorescence quenching.

To confirm direct binding of the compounds to Mcl-1, we have established a fluorescence-quenching assay based on the intrinsic Trp fluorescence of Mcl-1 [37]. Using this assay we have confirmed direct binding of marinopyrrole analogue **9** to Mcl-1 by generating binding isotherms and calculated the binding constant for **9** (K_d_ = 2.7 μM, Figure 3), consistent with its IC_50_ value in the ELISA assay.

### 2.5. Activity in Intact Human Breast Cancer Cells

**Figure 4:**
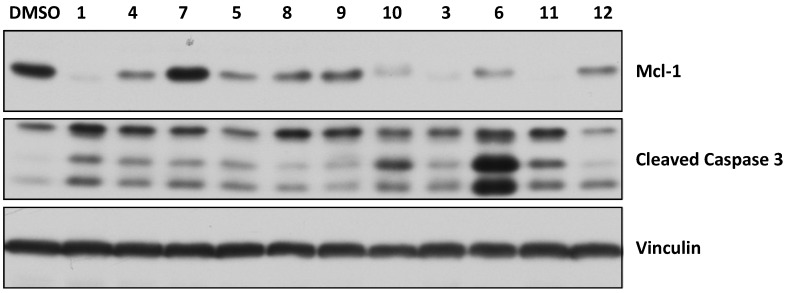
Effect of marinopyrroles on Mcl-1 levels in human breast cancer cells.

To determine if the marinopyrroles are active in intact cells, the human breast cancer MDA-MB-468 cells were treated with the marinopyrrole derivatives (40 μM for 16 h). The cells were then processed for Western blotting exactly as described by us previously [16,38]. Figure 4 shows that treatment of the cells with **1** resulted in a significant decrease in the levels of Mcl-1. This is consistent with our previous reports [10,16]. Figure 4 also shows that **3**, **4**, **5**, **6**, **10** and **11** were highly potent, whereas **8**, **9** and **12** were moderately active, at decreasing Mcl-1 levels. We next evaluated the effects of the compounds on programed cell death (apoptosis) by determining their ability to induce cleaved caspase 3 in MDA-MB-468 cells. Figure 4 shows that **1**, **3**, **4**, **5**, **6**, **7**, **10** and **11** potently induced apoptosis, whereas the ability of **8**, **9** and **12** to induce apoptosis was weak. None of the marinopyrroles affected the cellular levels of the control protein vinculin (Figure 4). In addition, the ability of the compounds to inhibit tumor growth of MDA-MB-468 cells was determined by MTT assays. To this end, MDA-MB-468 cells were treated for 48 h with the compounds at 0, 0.5, 5 and 50 μM, and the cells were processed for MTT assays as described by us [39]. All compounds inhibited MDA-MB-468 cell growth with **1**, **3** and **11** being the most potent with estimated IC_50_ values of 2, 3 and 2 μM, respectively. Compounds **4**, **5**, **6**, **7**, **8**, **9**, **10** and **12** were less potent and had estimated IC_50_ values of 28, 50, 50, 20, 50, 29, 20 and 16 μM, respectively. Finally, the effects of the compounds on nuclear and cellular morphology were assessed by DAPI nuclear staining and phase imaging. Figure S1 in Supplementary Information shows that the compounds had little effects on nuclear and cellular morphology.

## 3. Experimental Section

### 3.1. Synthesis of Marinopyrrole Derivatives

All chemicals were purchased from commercial suppliers and used without further purification. All solvents were dried and distilled before use. Tetrahydrofuran was distilled from sodium/benzophenone. Dichloromethane and acetonitrile were distilled over calcium hydride. Flash column chromatography was performed with silica gel (200–300 mesh). ^1^H NMR spectra were recorded at either 400 MHz or 600 MHz at ambient temperature. ^13^C NMR spectra were recorded at either 100 or 150 MHz at ambient temperature. Infrared spectra were recorded on a Perkin-Elmer Spectrum 100 spectrometer. Copies of the NMR spectra of all the described compounds are provided in an Electronic Supporting Information (ESI) document. Melting points were determined with a melting point apparatus (Fukai X-4). High resolution mass spectra were performed by electrospray ionization (ESI) on an Agilent ESI-TOF LC-MS 6200 system. Analytical HPLC was performed on an Agilent 1100 series instrument with diode array detectors and auto samplers. All tested compounds possessed a purity of not less than 95%.

(4,4′,5,5′-Tetrachloro-1′*H*-1,3′-bipyrrole-2,2′-diyl)bis(((2-hydroxy-4-(β-ethylacetate)thiophenyl) methanone) (**3**). Under N_2_, a mixture of **2** [14] (50 mg, 0.06 mmol), ethyl 2-mercaptoacetate (33 mg, 0.24 mmol), Pd_2_(dba)_3_ (2 mg, 0.003 mmol), xantphos (4 mg, 0.006 mmol) and *i*-Pr_2_NEt (31 mg, 0.24 mmol) was dissolved in 1,4-dioxane (5 mL). The reaction was heated to reflux and stirred for about 10 h. The reaction was quenched with water (10 mL) and extracted with EtOAc (15 mL × 3). The combined organic layers were dried over anhydrous sodium sulfate, filtered and concentrated in vacuum. The residue was purified by flash column chromatography (33% EtOAc/petroleum ether, *R*_f_ = 0.3) to yield **3** (28 mg, 61%) as a yellow solid. mp 51.8–53.3 °C; ^1^H NMR (400 MHz, CDCl_3_) δ 1.24–1.29 (m, 6H), 3.71 (s, 2H), 3.76 (s, 2H), 4.19–4.25 (m, 4H), 6.11 (s, 1H), 6.46 (d, *J* = 8.4 Hz, 1H), 6.73 (d, *J* = 8.0 Hz, 1H), 6.80 (d, *J* = 9.6 Hz, 2H), 7.20 (*br* s, 1H), 7.37 (*br* s, 1H), 10.51 (*br* s, 1H), 10.93 (s, 1H), 11.58 (s, 1H) ppm; ^13^C NMR (CDCl_3_, 100 MHz) δ 14.01, 14.01, 33.92, 34.17, 62.05, 62.09, 108.68, 111.90, 113.64, 114.13, 116.27, 116.27, 116.47, 116.80, 116.93, 118.74, 121.32, 122.17, 124.17, 124.62, 130.62, 133.37, 147.17, 148.83, 161.82, 162.94, 168.61, 168.70, 185.14, 186.83 ppm; HRMS ESI [M + H]^+^ calcd for C_30_H_25_Cl_4_N_2_O_8_S_2_ 744.9806, found 744.9812; IR (KBr) 3671, 3368, 3080, 2960, 2920, 1919, 1735, 1618, 1585, 1439, 1216, 1145, 1030, 745, 703 cm^−^^1^. HPLC purity, 96.8% (Flow rate: 1.0 mL/min; Column: Agilent ZORBAX 300SB-C8, 5 μm, 150 × 4.6 mm; Wavelength: UV 254 nm; Temperature: 25 °C; Mobile phase: MeOH:H_2_O = 90:10; *t*_R_ = 20.0 min).

(4,4′,5,5′-Tetrachloro-1′*H*-1,3′-bipyrrole-2,2′-diyl)bis(((2-hydroxy-4-benzylthio)phenyl)methanone) (**4**). Under N_2_, a mixture of **2** [14] (50 mg, 0.06 mmol), phenylmethanethiol (32 mg, 0.24 mmol), Pd_2_(dba)_3_ (2 mg, 0.003 mmol), Xantphos (4 mg, 0.006 mmol) and *i*-Pr_2_NEt (31 mg, 0.24 mmol) was dissolved in 1,4-dioxane (5 mL). The reaction was heated to reflux and stirred for about 4 h. The reaction was quenched with water (10 mL) and extracted with EtOAc (15 mL × 3). The combined organic layers were dried over anhydrous sodium sulfate, filtered and concentrated in vacuum. The residue was purified by flash column chromatography (15% EtOAc/petroleum ether, *R*_f_ = 0.3) to yield **4** (45 mg, 96%) as a yellow solid. mp 82.4–83.7 °C; ^1^H NMR (400 MHz, CDCl_3_) δ 4.17 (s, 2H), 4.22 (s, 2H), 6.11 (s, 1H), 6.47 (d, *J* = 8.4 Hz, 1H), 6.61 (*br*, s, 1H), 6.74 (s, 1H), 6.80 (s, 1H), 7.27–7.41 (m, 12H), 9.90 (*br* s, 1H), 10.95 (s, 1H), 11.63 (s, 1H) ppm; ^13^C NMR (CDCl_3_, 100 MHz) δ 36.24, 36.43, 108.77, 111.98, 113.35, 113.85, 115.84, 116.15, 116.26, 116.79, 117.01, 121.27, 122.15, 124.21, 124.72, 127.65, 127.65, 127.68, 127.68, 128.68, 128.68, 128.68, 128.78, 128.78, 128.78, 128.78, 130.46, 133.14, 135.34, 135.49, 149.49, 151.08, 161.84, 163.04, 185.08, 186.71 ppm; HRMS ESI [M + H]^+^ calcd for C_36_H_25_Cl_4_N_2_O_4_S_2_ 753.0010, found 753.0005; IR (KBr) 3410, 3236, 3061, 3028, 2923, 1616, 1581, 1483, 1448, 1391, 1327, 1223, 1073, 928, 780 cm^−^^1^. HPLC purity, 96.9% (Flow rate: 1.0 mL/min; Column: Agilent ZORBAX 300SB-C8, 5 μm, 150 × 4.6 mm; Wavelength: UV 254 nm; Temperature: 25 °C; Mobile phase: MeOH:H_2_O = 95:5; *t*_R_ = 6.6 min).

(4,4′,5,5′-Tetrachloro-1′*H*-1,3′-bipyrrole-2,2′-diyl)bis(((2-hydroxy-4-(4-methoxybenzylthio)phenyl)methanone) (**5**). Under N_2_, a mixture of **2** [14] (20 mg, 0.02 mmol), (4-methoxyphenyl) methanethiol (15 mg, 0.10 mmol), Pd_2_(dba)_3_ (0.8 mg, 0.001 mmol), Xantphos (1.6 mg, 0.002 mmol) and *i*-Pr_2_NEt (12 mg, 0.10 mmol) was dissolved in 1,4-dioxane (3 mL). The reaction was heated to reflux and stirred for about 4 h. The reaction was quenched with water (10 mL) and extracted with EtOAc (10 mL × 3). The combined organic layers were dried over anhydrous sodium sulfate, filtered and concentrated in vacuum. The residue was purified by flash column chromatography (20% EtOAc/petroleum ether, *R*_f_ = 0.3) to yield **5** (17 mg, 85%) as a yellow solid. mp 85.4–86.7 °C; ^1^H NMR (400 MHz, CDCl_3_) δ 3.78 (s, 3H), 3.79 (s, 3H), 4.13 (s, 2H), 4.18 (s, 2H), 6.11 (s, 1H), 6.46 (d, *J* = 8.4 Hz, 1H), 6.62 (s, 1H), 6.74 (s, 1H), 6.80 (s, 1H), 6.86 (dd, *J* = 8.4, 3.2 Hz, 5H), 7.31 (t,* J* = 8.4 Hz, 5H), 9.71 (*br* s, 1H), 10.99 (s, 1H), 11.64 (s, 1H) ppm; ^13^C NMR (CDCl_3_, 100 MHz) δ 35.72, 35.90, 53.66, 55.25, 108.83, 111.94, 113.30, 113.83, 114.18, 114.18, 114.18, 114.18, 114.18, 115.78, 116.11, 116.24, 116.72, 117.01, 121.23, 121.96, 124.08, 124.77, 127.11, 127.29, 129.85, 129.85, 129.96, 129.96, 130.40, 133.12, 149.68, 151.31, 159.01, 159.06, 161.90, 163.05, 185.02, 186.71 ppm; HRMS ESI [M + H]^+^ calcd for C_38_H_29_Cl_4_N_2_O_6_S_2_ 813.0221, found 813.0228; IR (KBr) 3421, 3253, 2929, 2836, 1702, 1615, 1511, 1447, 1393, 1329, 1248, 1177, 1075, 1034, 943 cm^−^^1^. HPLC purity, 96.5% (Flow rate: 1.0 mL/min; Column: Agilent ZORBAX 300SB-C8, 5 μm, 150 × 4.6 mm; Wavelength: UV 254 nm; Temperature: 25 °C; Mobile phase: MeOH:H_2_O = 95:5; *t*_R_ = 6.4 min).

(4,4′,5,5′-Tetrachloro-1′*H*-1,3′-bipyrrole-2,2′-diyl)bis(((2-hydroxy-4-(β-ethylacetate)sulfonylphenyl) methanone) (**6**). To a solution of **3** (22 mg, 0.03 mmol) in CH_2_Cl_2_ (2 mL) was added a solution of *m*-CPBA (31 mg, 0.18 mmol) in CH_2_Cl_2_ (1 mL) at room temperature. After being stirred for about 20 h, the reaction was quenched by addition water (10 mL) and extracted with CH_2_Cl_2_ (10 mL × 3). The combined organic layers were dried over anhydrous sodium sulfate, filtered and concentrated in vacuum. The residue was purified by flash column chromatography (20% acetone, 27% EtOAc, 53% petroleum ether, *R*_f_ = 0.2) to yield **6** (16.7 mg, 70%) as a yellow solid. mp 90.0–91.7 °C; ^1^H NMR (400 MHz, acetone-*d*_6_) δ 1.16–1.18 (m, 6H), 4.09–4.14 (m, 4H), 4.33 (s, 2H), 4.42 (s, 2H), 6.48 (s, 1H), 7.32 (d, *J* = 8.0 Hz, 1H), 7.45–7.52 (m, 3H), 7.61 (d, *J* = 8.0 Hz, 1H), 7.23 (d, *J* = 8.0 Hz, 1H), 10.48 (*br* s, 2H), 12.33 (*br* s, 1H) ppm; ^13^C NMR (acetone-*d*_6_, 100 MHz) δ 14.14, 14.14, 60.98, 61.02, 62.58, 62.58, 110.42, 112.21, 117.11, 117.38, 118.94, 119.29, 119.65, 122.89, 124.02, 126.06, 127.21, 128.65, 128.75, 129.08, 131.08, 132.87, 144.04, 144.64, 157.98, 158.59, 163.00, 163.05, 183.41, 184.48 ppm; HRMS ESI [M + H]^+^ calcd for C_30_H_25_Cl_4_N_2_O_12_S_2_ 808.9603, found 808.9599; IR (KBr) 2918, 2850, 2490, 1741, 1637, 1589, 1441, 1407, 1330, 1269, 1215, 1151, 1029, 751, 703, 637 cm^−^^1^. HPLC purity, 98.1% (Flow rate: 1.0 mL/min; Column: Waters C18, 5 μm, 150 × 4.6 mm; Wavelength: UV 254 nm; Temperature: 25 °C; Mobile phase: MeOH:H_2_O = 70:30; *t*_R_ = 30.4 min).

(4,4′,5,5′-Tetrachloro-1′*H*-1,3′-bipyrrole-2,2′-diyl)bis(((2-hydroxy-4-benzylsulfonyl)phenyl)methanone) (**7**). To a solution of **4** (30 mg, 0.04 mmol) in CH_2_Cl_2_ (2 mL) was added a solution of *m*-CPBA (42 mg, 0.24 mmol) in CH_2_Cl_2_ (1 mL) at room temperature. After being stirred for about 5 h, the reaction was quenched by addition water (10 mL) and extracted with CH_2_Cl_2_ (10 mL × 3). The combined organic layers were dried over anhydrous sodium sulfate, filtered and concentrated in vacuum. The residue was purified by flash column chromatography (20% acetone, 27% EtOAc, 53% petroleum ether, *R*_f_ = 0.2) to yield **7** (24 mg, 75%) as a yellow solid. mp 130.9–132.7 °C; ^1^H NMR (400 MHz, CDCl_3_) δ 4.26 (s, 2H), 4.34 (s, 2H), 6.12 (s, 1H), 6.99 (d, *J* = 8.4 Hz, 1H), 7.05 (d, *J* = 8.4 Hz, 1H), 7.14–7.17 (m, 4H), 7.29–7.37 (m, 8H), 7.58 (d, *J* = 8.4 Hz, 1H), 7.68 (d, *J* = 8.0 Hz, 1H) ppm; ^13^C NMR (CDCl_3_, 100 MHz) δ 62.17, 62.35, 110.30, 110.34, 112.10, 117.23, 117.51, 118.83, 119.18, 119.80, 124.03, 126.02, 127.12, 127.83, 128.40, 128.75, 129.07, 129.15, 129.15, 129.19, 129.19, 129.34, 129.40, 129.45, 131.25, 131.88, 131.88, 131.88, 131.98, 131.98, 132.82, 144.07, 144.21, 158.24, 183.74, 184.70 ppm; HRMS ESI [M + H]^+^ calcd for C_36_H_25_Cl_4_N_2_O_8_S_2_ 816.9806, found 816.9800; IR (KBr) 3078, 3030, 2959, 2920, 2851, 2583, 1730, 1636, 1591, 1142, 1407, 1319, 1149, 1125, 879, 750, 701, 628 cm^−^^1^. HPLC purity, 97.1% (Flow rate: 1.0 mL/min; Column: Agilent ZORBAX 300SB-C8, 5 μm, 150 × 4.6 mm; Wavelength: UV 254 nm; Temperature: 25 °C; Mobile phase: MeOH:H_2_O = 60:40; *t*_R_ = 11.9 min).

(4,4′,5,5′-Tetrachloro-1′*H*-1,3′-bipyrrole-2,2′-diyl)bis(((2-hydroxy-4-(4-methoxybenzylsulfonyl)phenyl) methanone) (**8**). To a solution of **5** (60 mg, 0.07 mmol) in CH_2_Cl_2_ (5 mL) was added a solution of *m*-CPBA (128 mg, 0.74 mmol) in CH_2_Cl_2_ (2 mL) at room temperature. After being stirred for about 20 h, the reaction was quenched by adding water (10 mL) and extracted with CH_2_Cl_2_ (10 mL × 3). The combined organic layers were dried over anhydrous sodium sulfate, filtered and concentrated in vacuum. The residue was purified by flash column chromatography (20% acetone, 27% EtOAc, 53% petroleum ether, *R*_f_ = 0.2) to yield **8** (42 mg, 65%) as a yellow solid. mp 104.3–106.3 °C; ^1^H NMR (400 MHz, CDCl_3_) δ 3.77 (s, 6H), 4.20 (s, 2H), 4.31 (s, 2H), 6.17 (s, 1H), 6.80 (t, *J* = 7.2 Hz, 4H), 6.97 (d, *J* = 8.4 Hz, 1H), 7.05 (d, *J* = 8.4 Hz, 5H), 7.16 (s, 1H), 7.36 (s, 1H), 7.62 (d, *J* = 8.4 Hz, 1H), 7.67 (d, *J* = 8.0 Hz, 1H), 10.44 (*br* s, 1H), 11.00 (*br* s, 1H) ppm; ^13^C NMR (CDCl_3_, 100 MHz) δ 55.26, 55.26, 61.84, 61.84, 109.45, 112.63, 114.18, 114.18, 114.18, 114.22, 114.22, 117.59, 118.33, 118.57, 118.57, 118.82, 118.93, 122.16, 122.80, 123.45, 124.62, 125.25, 126.44, 131.09, 132.02, 132.02, 132.02, 132.09, 132.09, 133.78, 143.91, 144.84, 160.15, 160.22, 160.63, 161.77, 184.85, 187.06 ppm; HRMS ESI [M + Na]^+^ calcd for C_38_H_28_Cl_4_N_2_NaO_10_S_2_ 898.9837, found 898.9838; IR (KBr) 2961, 2920, 2850, 1730, 1632, 1592, 1512, 1444, 1257, 1148, 1099, 1030, 798 cm^−^^1^. HPLC purity, 95.1% (Flow rate: 1.0 mL/min; Column: Agilent ZORBAX 300SB-C8, 5 μm, 150 × 4.6 mm; Wavelength: UV 254 nm; Temperature: 25 °C; Mobile phase: MeOH:H_2_O = 65:35; *t*_R_ = 6.0 min).

(4,4′,5,5′-Tetrachloro-1′*H*-1,3′-bipyrrole-2,2′-diyl)bis(((2-hydroxy-4-(β-acetic acid)thiophenyl) methanone) (**9**). To a solution of **3** (20 mg, 0.03 mmol) in a mixture of H_2_O/THF (1:2, 3 mL) was added LiOH (15 mg, 0.35 mmol) at room temperature. The reaction was allowed to warm up to 50 °C and stirred for about 10 h. The reaction was adjusted to pH 5.0 with 0.5 N HCl and extracted with EtOAc (5 mL × 3). The combined organic layers were dried over anhydrous sodium sulfate, filtered and concentrated in vacuum. The residue was purified by reverse-phase column chromatography (C18 reverse silica gel, 7% AcOH, 22% H_2_O, 71% MeOH, *R*_f_ = 0.2) to yield **9** (15.7 mg, 85%) as a brown solid. mp 137.9–139.7 °C; ^1^H NMR (400 MHz, DMSO-*d*_6_) δ 3.46 (s, 2H), 3.65 (s, 2H), 6.03 (s, 1H), 6.53–6.60 (m, 3H), 6.64 (s, 1H), 7.38 (d, *J* = 7.6 Hz, 1H), 7.85 (d, *J* = 8.0 Hz, 1H) ppm; ^13^C NMR (DMSO-*d*_6_, 100 MHz) δ 36.10, 36.80, 108.30, 109.39, 112.71, 112.94, 115.56, 116.02, 116.84, 118.22, 120.95, 122.60, 123.09, 124.39, 128.56, 131.13, 132.65, 133.61, 144.10, 149.87, 159.26, 160.70, 171.50, 172.90, 181.57, 186.55 ppm; HRMS ESI [M + Na]^+^ calcd for C_26_H_16_Cl_4_N_2_NaO_8_S_2_ 710.9000, found 710.9009; IR (KBr) 3392, 2955, 2918, 2849, 1592, 1382, 1223, 1023, 671 cm^−^^1^. HPLC purity, 98.7% (Flow rate: 1.0 mL/min; Column: Agilent ZORBAX 300SB-C8, 5 μm, 150 × 4.6 mm; Wavelength: UV 254 nm; Temperature: 25 °C; Mobile phase: MeOH:H_2_O = 60:40; *t*_R_ = 8.0 min).

(4,4′,5,5′-Tetrachloro-1′*H*-1,3′-bipyrrole-2,2′-diyl)bis(((2-hydroxy-4-(β-acetic acid)sulfonylphenyl)methanone) **(10**). To a solution of **6** (70 mg, 0.09 mmol) in a mixture of H_2_O/THF (1:2, 5 mL) was added LiOH (49 mg, 1.13 mmol) at room temperature. The reaction was allowed to warm up to 50 °C and stir for about 3 h. The reaction was adjusted to pH 5.0 with 0.5 N HCl and extracted with EtOAc (10 mL × 3). The combined organic layers were dried over anhydrous sodium sulfate, filtered and concentrated in vacuum. The residue was purified by reverse column chromatography (C18 reverse silica gel, 4% AcOH, 38% H_2_O, 58% MeOH, *R*_f_ = 0.2) to yield **10** (62 mg, 95%) as a yellow solid. mp 143.2–144.7 °C; ^1^H NMR (400 MHz, DMSO-*d*_6_) δ 4.12 (s, 2H), 4.18 (s, 2H), 6.22 (s, 1H), 7.15 (d, *J* = 8.0 Hz, 1H), 7.27–7.29 (m, 4H), 7.36–7.40 (m, 1H) ppm; ^13^C NMR (acetone-*d*_6_, 100 MHz) δ 52.09, 52.09, 100.10, 100.18, 100.98, 105.79, 107.20, 108.26, 108.65, 112.73, 113.40, 115.44, 118.90, 118.91, 120.25, 120.40, 120.53, 121.64, 133.09, 146.18, 146.89, 154.58, 163.09, 163.09, 171.58, 172.34 ppm; HRMS ESI [M + Na]^+^ calcd for C_26_H_17_Cl_4_N_2_O_12_S_2_ 752.8977, found 752.8981; IR (KBr) 3395, 2957, 2923, 1628, 1445, 1407, 1313, 1147, 1026, 1000, 906, 825, 701 cm^−^^1^. HPLC purity, 95.2% (Flow rate: 1.0 mL/min; Column: Agilent ZORBAX 300SB-C8, 5 μm, 150 × 4.6 mm; Wavelength: UV 254 nm; Temperature: 25 °C; Mobile phase: MeOH:H_2_O = 60:40; *t*_R_ = 4.0 min).

### 3.2. Fluorescence Quenching, Enzyme-Linked Immunosorbent Assay (ELISA) and Western Blotting Following Treatment of Intact Human Breast Cancer Cells

#### 3.2.1. Fluorescence Quenching

Recombinant His-tagged Mcl-1 (residues 172–327) was purified as previously described [37]. Intrinsic Trp fluorescence of Mcl-1 was measured with a TECAN M1000 plate reader at excitation wavelength of 285 nm and emission wavelengths of 310–400 nm. Serial dilutions of compound **9** (starting from 20 μM) in 20 mM K-phosphate pH 7.6, 150 nM KCl was used to assay compound binding using 2.5 μM of Mcl-1. Each condition is run at triplicate and binding constants are calculated by nonlinear regression analysis of dose response curves using Prism software 6.0 (Graphpad, La Jolla, CA, USA). Control experiments included titration of DMSO solvent and fluorescence quenching of denatured Mcl-1 in the presence of compound.

#### 3.2.2. Enzyme-Linked Immunosorbent Assay

ELISAs were performed using a similar procedure as previously described [16]. Briefly, 40 nM of biotinylated Bim BH3 peptide (Biomatik, Wilmington, DE, USA) in SuperBlock blocking buffer (Thermo Scientific Pierce, Rockford, IL, USA) was incubated in high-binding capacity streptavidin coated plates (Thermo Scientific Pierce, Rockford, IL, USA) for 2 h. Compounds were diluted in 120 µL of PBS containing 10 nM of GST-Mcl-1 or 10 nM GST-Bcl-xL in 1.5-mL tubes for 15 min. Wells were washed with wash buffer (PBS containing 0.05% Tween-20) and then 100 µL of the compound/GST-protein mixture was transferred to the Bim-containing wells. The plates were incubated for 2 h, and then, the wells were washed with wash buffer. HRP-conjugated anti-GST antibody (Bethyl Laboratories, Montgomery, TX, USA) was diluted 1:2000 in SuperBlock, and 100 µL were transferred to each well. The plate was incubated for 1 h, and then, the wells were washed with wash buffer followed by PBS. One hundred microliters of SureBlue TMB Microwell Peroxidase Substrate (VWR International, Radnor, PA, USA) was added to each well, and the plates were developed for 5–10 min. One hundred microliters of 1 N HCl was added to each well to stop the reaction, and the absorbance was read at 450 nm using a µQuant plate reader (Bio-Tek Instruments, Winooski, VT, USA).

#### 3.2.3. Western Blotting Following Treatment of Intact Human Breast Cancer Cells

Treatment of the human breast cancer (MDA-MB-468) cells and Western blotting were performed using the methods described by us previously [16,38].

#### 3.2.4. MTT Assays, Nuclear and Cellular Morphology

MDA-MB-468 cells were treated and processed for MTT, DAPI nuclear staining and phase imaging of cells as described previously by us [39].

## 4. Conclusions

This article describes general synthetic routes to access novel marinopyrrole derivatives with sulfide and sulphone spacers and an evaluation of their *in vitro* activity against the binding of the pro-survival proteins, Mcl-1 and Bcl-xL, to the pro-apoptotic protein, Bim. Our efforts were focused on improving anti-Mcl-1/Bim and Bcl-xL/Bim potency and selectivity. SAR studies of these marinopyrrole derivatives have clearly demonstrated that: (i) symmetrical marinopyrrole **3** with ethyl mercaptoacetate extended in the *para-*position to the carbonyl group with a sulfide spacer improved the potency by five- and 17.6-fold (**3** *vs.* **1**) for disrupting Mcl-1/Bim and Bcl-xL/Bim binding, respectively (Figure 2); (ii) symmetrical marinopyrroles with CH_2_Ph and/or CH_2_(*p-*MeOPh) extended in the *para-*position to the carbonyl group with sulfide spacers (**4** and **5**) are not only 12.7-fold and 27.3-fold more potent than the parent marinopyrrole **1** against Mcl-1/Bim and Bcl-xL/Bim but also potent dual inhibitors (IC_50_ = 0.6 µM and 0.7 µM, respectively); (iii) Potency was decreased dramatically when the sulfide was replaced by sulphone spacers (Figure 2: **3** *vs.* **6**, **4** *vs.* **7** and **5** *vs.* **8**); (iv) Although marinopyrroles with sulphone spacers are less potent than their sulfide counterparts against both Mcl-1/Bim and Bcl-xL/Bim, they are in general more selective for Mcl-1/Bim over Bcl-xL/Bim (Figure 2); (v) the symmetrical marinopyrrole **9** with mercaptoacetic acid in the *para-*position to the carbonyl group is an excellent “lead” for further optimization of Mcl-1 selective inhibitors (IC_50_ = 6.1 µM, 16.4-fold more selective for Mcl-1 over Bcl-xL, Figure 2); (vi) nonsymmetrical marinopyrrole **12** exhibited the same potency as **1** against both Mcl-1/Bim and Bcl-xL/Bim. Furthermore, most of the derivatives were cell active as demonstrated by their ability to decrease the levels of Mcl-1, induce apoptosis and inhibit tumor cell growth of human breast cancer cells. In summary, we have designed and synthesized a series of novel symmetrical and nonsymmetrical marinopyrroles with improved potency against both Mcl-1 and Bcl-xL. Further optimization is actively ongoing, and the activity, selectivity and absorption, distribution, metabolism and excretion (ADME)/tox data of these compounds will be published in due course.

## Supplementary Files

Supplementary File 1

## Abbreviations

ADME: absorption, distribution, metabolism and excretion
Bcl-2: B-cell lymphoma 2
Bcl-xL: B-cell lymphoma extra large
BH3: Bcl-2 homology domain 3
calcd.: calculated
*m*-CPBA: *m*-chloroperbenzoic acid
DCM: dichloromethane
dd: double doublet
*br*: broad
DPPP: bis(diphenylphosphino)propane
DIEA: diisopropylethylamine
DMF: dimethylformamide
DMSO: dimethyl sulfoxide
EtOAc: ethyl acetate
ESI: electrospray ionization
HPLC: high performance liquid chromatography
HRMS: high resolution mass spectrometry
HRP: horseradish peroxidase
IR: infrared
KBr: potassium bromide
KF: potassium fluoride
LC: liquid chromatography
LiOH: lithium hydroxide
Mcl-1: Myeloid cell leukemia 1
MeCN: acetonitrile
MeOH: methyl alcohol
MDA-MB-468: breast cancer cell line
mp: melting point
MRSA: methicillin-resistant *Staphylococcus aureus*
NCS: *N*-chlorosuccinimide
NaI: sodium iodide
NMR: nuclear magnetic resonance
PBS: phosphate-buffered saline
RO5: Rule of Five
SAR: structure activity relationship; s, singlet
THF: tetrahydrofuran
TMB: 3,3′,5,5′-Tetramethylbenzidine;
tox: toxicity
Pd_2_(dba)_3_: tris(dibenzylideneacetone)dipalladium
