# Supplementary material for: Marinopyrrole Derivatives with Sulfide Spacers as Selective Disruptors of Mcl-1 Binding to Pro-Apoptotic Protein Bim
Source: Mar Drugs. 2014 Jul 29;12(8):4311–25. doi: 10.3390/md12084311 (PMC4145318; doi:10.3390/md12084311)
Supplement: Supplementary File 1 [file marinedrugs-12-04311-s001.pdf]

Supplementary Information

Figure S1. Effects of marinopyrroles on nuclear and cellular morphology.

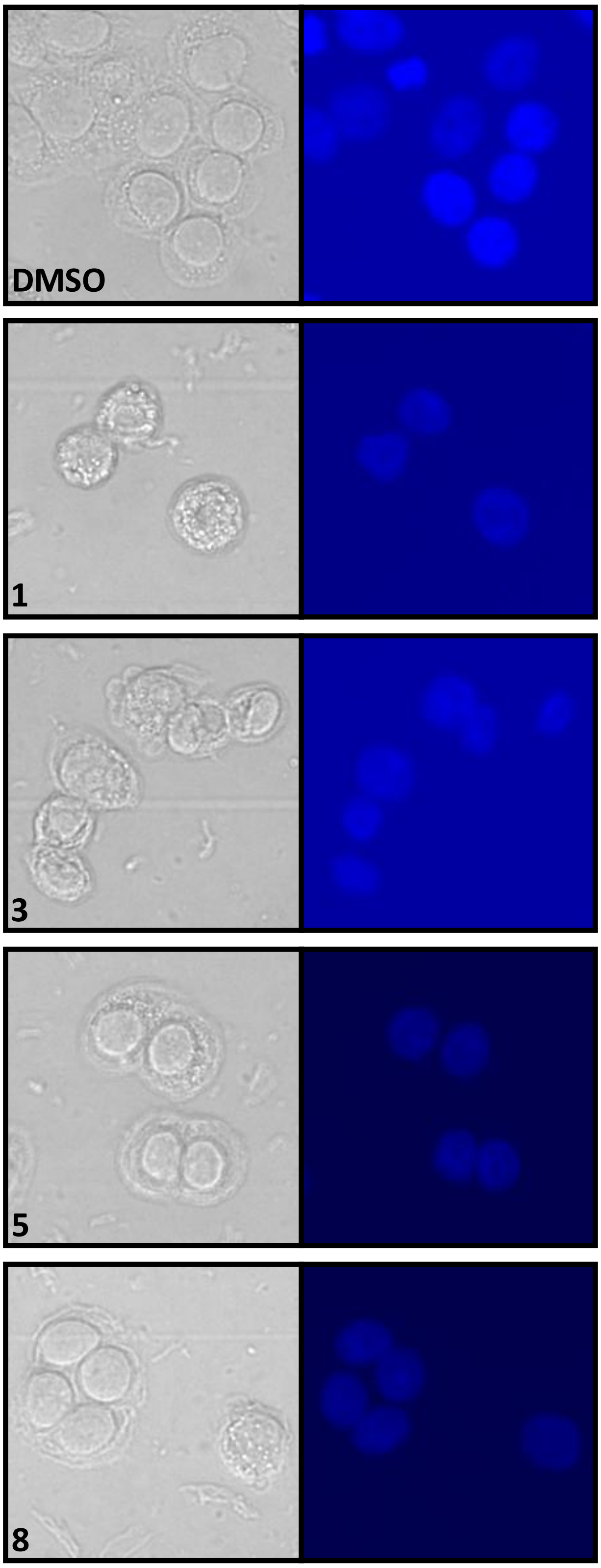

© 2014 by the authors; licensee MDPI, Basel, Switzerland. This article is an open access article distributed under the terms and conditions of the Creative Commons Attribution license (http://creativecommons.org/licenses/by/3.0/).
